# Supplementary material for: Change Rates and Prevalence of a Dichotomous Variable: Simulations and Applications
Source: PLoS One. 2015 Mar 6;10(3):e0118955. doi: 10.1371/journal.pone.0118955 (PMC4352043; doi:10.1371/journal.pone.0118955)
Supplement: S1 Supporting Information — Analytical steps to deduce Equation (3) from system (2). (DOC) [file pone.0118955.s001.doc]

# Derivation of the partial differential equation - analytical steps to deduce Equation (3) from system (2).

## Supporting Information 1 to *Change rates and prevalence of a dichotomous variable: simulations and applications* by Ralph Brinks and Sandra Landwehr.

In the following we shortly write ∂*_x_ = ∂ /* ∂*x* for the partial derivative with respect to the variable *x* ∈{*t, a*}. With the assumption that the numbers *S = S*(*t, a*) and *C = C*(*t, a*) of susceptible and diseased individuals are partially differentiable functions, the change rates are given by the system

(∂*_t_* + ∂*_a_*) *S* = – [*i* + *m*_A_] *S* + *r* *C,* (A.1)

(∂*_t_* + ∂*_a_*) *C* = *i* *S* – [*m*_B_ + *r*] *C.* (A.2)

This is a system of partial differential equations (PDEs). For the case of no remission, i.e. *r=0*, the PDE (A.1) with initial condition *S*(*t,* 0) *= S*_0_(*t*) describing the number of healthy new-borns at calendar time *t* has a solution of the form

$S(t,a)=S_{0}(t-a) \exp\left(-\int_{0}^{a} \left[m_{A}(t-a+s,s)+i(t-a+s,s)\right] ds\right)$.

Analogously, the PDE (A.2) with initial condition *C*(*t,* 0) *=* 0 (we just consider diseases contracted after birth) is solved by

$$C(t,a)=\int_{0}^{a} i(t-s,a-s) S(t-s,a-s) \exp\left(-\int_{0}^{s} (m_{B}(t-a+\varsigma,\varsigma) d\varsigma\right) ds,$$

which can be shown using the method of characteristics (Polyanin AD, Zaitsev VF, Moussiaux A: Handbook of First-Order Partial Differential Equations, CRC Press 2001)

We use equations (A.1) and (A.2) to obtain a one-dimensional PDE describing the temporal and age-related changes in the prevalence

$$p=p(t,a)=\frac{C(t,a)}{C(t,a)+S(t,a)}.$$

As we are interested in the change rate of the prevalence, we use the standard rules of differentiation to obtain

$$\begin{matrix} (\partial_{t}+\partial_{a}) p=\frac{(S+C) (\partial_{t}+\partial_{a}) C-C (\partial_{t}+\partial_{a}) (S+C) }{(S+C)^{2}} \\ =\frac{(S+C) \left[i S-(m_{B}+r) C\right]-C \left[i S-(m_{B}+r) C-(i+m_{A}) S+r C\right] }{(C+S)^{2}} \\ =\frac{i S-(m_{B}+r) C}{S+C}-\frac{C \left[-m_{B} C-m_{A} S\right]}{(S+C)^{2}} \\ =i \frac{S}{S+C}-(m_{B}+r) \frac{C}{S+C}+m_{B} \left(\frac{C}{S+C}\right)^{2}+m_{A} \frac{S C}{(S+C)^{2}} \\ =i (1-p)-(m_{B}+r) p+m_{B} p^{2}+m_{A} p (1-p) \\ =(1-p) \left[i+m_{A} p-m_{B} p\right]-r p \\ =(1-p) \left[i-p \left(m_{B}-m_{A}\right)\right]-r p \end{matrix}$$

via elementary calculus.
